# Optical engineering of infrared PbS CQD photovoltaic cells for wireless optical power transfer systems

**DOI:** 10.1007/s12200-023-00069-0

**Published:** 2023-06-15

**Authors:** Mengqiong Zhu, Yuanbo Zhang, Shuaicheng Lu, Zijun Wang, Junbing Zhou, Wenkai Ma, Ruinan Zhu, Guanyuan Chen, Jianbing Zhang, Liang Gao, Jiancan Yu, Pingqi Gao, Jiang Tang

**Affiliations:** 1 School of Materials, Shenzhen Campus of Sun Yat-Sen University, Shenzhen, 518107 China; 2 Wuhan National Laboratory for Optoelectronics (WNLO) and School of Optical and Electronic Information, Huazhong University of Science and Technology (HUST), Wuhan, 430074 China

**Keywords:** Wireless optical power transfer, PbS CQDs, Photovoltaic cells, Optical engineering

## Abstract

Infrared photovoltaic cells (IRPCs) have attracted considerable attention for potential applications in wireless optical power transfer (WOPT) systems. As an efficient fiber-integrated WOPT system typically uses a 1550 nm laser beam, it is essential to tune the peak conversion efficiency of IRPCs to this wavelength. However, IRPCs based on lead sulfide (PbS) colloidal quantum dots (CQDs) with an excitonic peak of 1550 nm exhibit low short circuit current (*J*_sc_) due to insufficient absorption under monochromatic light illumination. Here, we propose comprehensive optical engineering to optimize the device structure of IRPCs based on PbS CQDs, for 1550 nm WOPT systems. The absorption by the device is enhanced by improving the transmittance of tin-doped indium oxide (ITO) in the infrared region and by utilizing the optical resonance effect in the device. Therefore, the optimized device exhibited a high short circuit current density of 37.65 mA/cm^2^ under 1 sun (AM 1.5G) solar illumination and 11.91 mA/cm^2^ under 1550 nm illumination 17.3 mW/cm^2^. Furthermore, the champion device achieved a record high power conversion efficiency (PCE) of 7.17% under 1 sun illumination and 10.29% under 1550 nm illumination. The PbS CQDs IRPCs under 1550 nm illumination can even light up a liquid crystal display (LCD), demonstrating application prospects in the future.

**Graphical Abstract:**

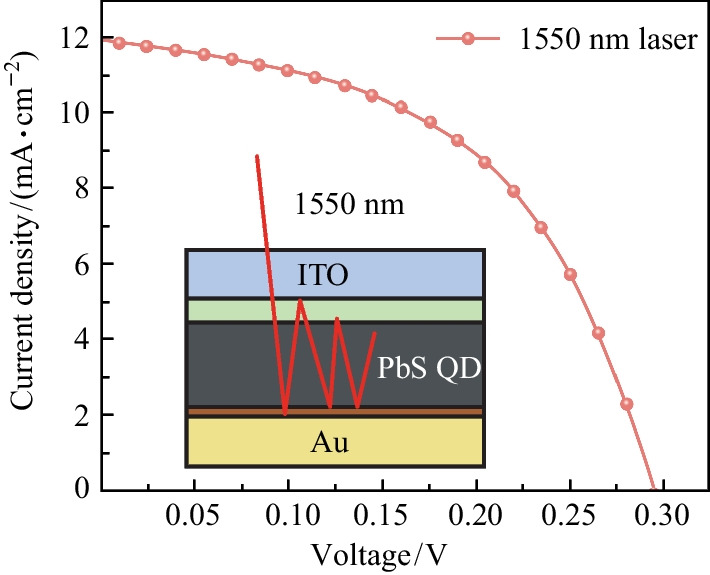

**Supplementary Information:**

The online version contains supplementary material available at 10.1007/s12200-023-00069-0.

## Introduction

**Fig. 1:**
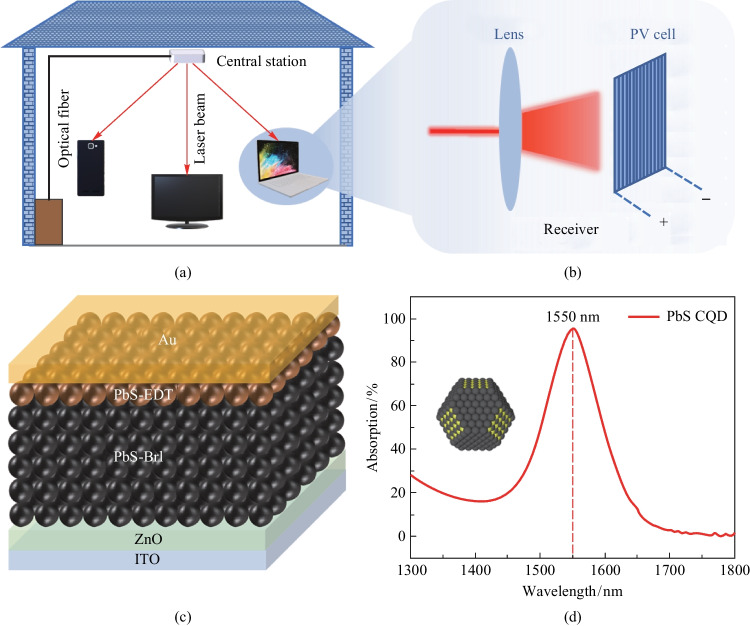
**a** Illustration of the WOPT technique, which is equipped with **b** IRPCs. **c** Device architecture of the PbS CQD IRPCs, and **d** the absorption spectrum of PbS CQD solution (2 mg/mL)

Infrared photovoltaic cells (IRPCs) are attracting interests due to their potential applications in wireless optical power transfer (WOPT) systems, which converts the infrared laser light into electric energy (Fig. 1a and b) [1, 2]. With the increasing potential to realize long-range wireless power transfer [2–5], WOPT technology has shown great application prospects in chargers of portable electronic devices [6], sensors in the Internet of Things [7], as well as devices in industrial environments in which assembling or replacing cables is difficult [8–11]. Laser beams utilized in the WOPT system tend to have an optical fiber communication wavelength of 1550 nm [1, 11], which is safer for human eyes and is able to integrate with optical fibers. The laser beam wavelength of 1550 nm determines that the optimal bandgap of photovoltaic materials for the WOPT system is about 0.8 eV. Yet the growth of prospective materials like GaSb [12, 13] (0.73 eV) and InGaAs [14] (0.36–0.75 eV) requires ultra-high vacuum equipment and lattice-matched substrates which leads to complexity in process and high production costs. In contrast, lead sulfide colloidal quantum dots are promising materials for 1550 nm IRPCs with the advantages of bandgap tuning, high absorption coefficient, and solution processing [15].

Current research on PbS CQD photovoltaic cells primarily focuses on full-spectrum solar cells and infrared solar cells pairing with silicon or perovskite cells [16–21]. Recent advances in surface passivation, device structure, and interface engineering have pushed the PCE of PbS CQD solar cells to reach 15.45% [22] and that of PbS CQD infrared solar cells to reach 11.98% [20]. Yet IRPCs required for fiber-based WOPT systems need utilize larger PbS QDs with a bandgap of ≈ 0.8 eV and a size of ≈ 5.9 nm. In the past few years, PbS CQD IRPCs with exciton absorption peaks at ~ 1550 nm, or longer wavelengths, have been developed as bottom subcells of thin-film III–V semiconductor tandem solar cells [23–26]. In 2018, the Sargent group built PbS QD solar cells using CQDs with a 0.8 eV bandgap and achieved a high external quantum efficiency (EQE) of 50% at 1550 nm [23]. In 2019, Konstantatos et al. reported photovoltaic cells using CQDs with exciton absorption peaks at 1620 nm that exhibit a high PCE of 6.4% under the full solar spectrum [26]. These devices are usually n-i-p structures with zinc oxide (ZnO) as the electron transport layer (ETL) and 1,2-ethanedithiol (EDT)-treated CQD as the hole transport layer (HTL). However, all of the reported PbS CQD IRPCs are designed to operate under the solar spectrum [17, 24, 26–28] whereas the absorption of IRPCs driven by monochromatic light is more sensitive to their structures. Thus, optical engineering is a vital strategy to enhance the performance of PbS-CQD-based IRPCs for fiber-based WOPT systems.

Here we developed high-performance IRPCs utilizing PbS CQDs with a peak absorption wavelength of 1550 nm. The device structure was optimized by improving ITO transmittance combined with the optical resonance effect. The champion IRPCs exhibit a high PCE of 10.29% under 1550 nm illumination (17.3 mW/cm^2^) and a high EQE of 51% at 1550 nm. Remarkably, this device achieved a record high PCE of 7.17% under 1 sun illumination, which is significantly higher than those reported devices that use PbS CQDs with an exciton absorption peak at 1550 nm. We further demonstrated the powering capacity of the PbS CQDs IRPCs by lighting an LCD under 1550 nm illumination.

## Results and discussion

PbS CQDs are synthesized by a colloidal method that allows for precise band-gap engineering by size manipulation through adjusting growth kinetics and thermodynamics [15, 18, 29, 30]. The peak absorption of PbS CQDs that were utilized as the active material in this work was tuned to 1550 nm by controlling the growth time (Fig. 1d and Fig. S1b). Moreover, we adopted a cation exchange method [18] to get monodispersed PbS CQDs with fewer exposed (100) facets; this is beneficial for charge transport in the CQD film [31–33]. In this method, the sulfur source was ZnS CQDs synthesized by a hot injection method (Fig. S1a) and PbS CQDs were formed by the exchange of zinc cations with Pb cations in the PbCl_2_ solution.

**Fig. 2:**
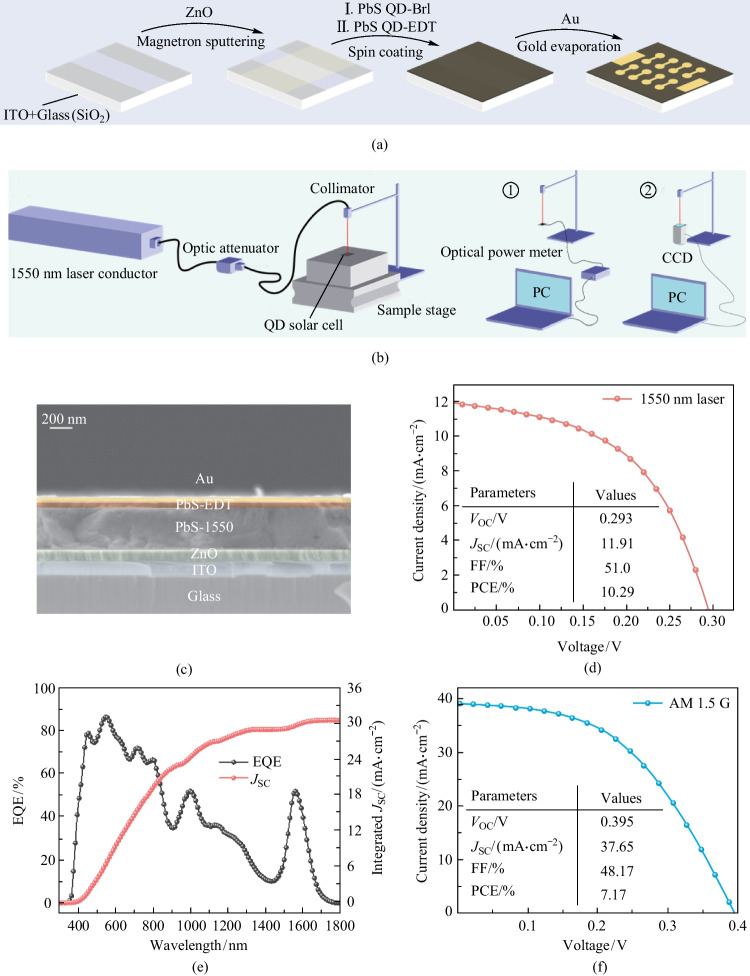
Fabrication and characterization of the IRPCs. **a** Overview of the device fabrication process. **b** System constructed to characterize the IRPC performance under 1550 nm illumination. **c** Cross-sectional SEM image of Glass/ITO/ZnO/PbS-1550/PbS-EDT/Au device. **d** Current–voltage (*J*–*V*) curves and performance parameters of the champion IRPC under 1550 nm illumination (17.3 mW/cm^2^). **e** EQE spectrum and integrated current density of the champion IRPC (no bias). **f** *J*–*V* curves and performance parameters of the champion IRPC under 1 sun illumination

We fabricated photovoltaic devices with an architecture of Glass/ITO/ZnO/PbS-1550/PbS-EDT/Au (Fig. 1c), and the device fabrication process is illustrated in Fig. 2a. We used commercial ITO as the transparent electrode and the ZnO layer deposited by magnetron sputtering as ETL for the reference devices. Then PbS CQD ink was prepared by the hybrid halide ligand strategy [19], where the native organic surface ligands were exchanged with halide anions of lead halides (PbI_2_:PbBr_2_ = 2.3:1). The QD ink was spin-coated onto the ZnO working as the photoactive layer. After that, a thin layer of EDT-treated PbS QDs with a bandgap of 1.4 eV acted as HTL. Finally, Au was deposited and worked as the top electrode.

To evaluate the PCE of the IRPCs under 1550 nm illumination, a system was self-constructed with a collimated 1550 nm fiber laser as the light source (Fig. 2b). Moreover, the system incorporated an optic attenuator to investigate the performance of the IRPC under different illumination power densities. Meanwhile, we designed a characterization scheme to determine both the power and the incident area of the laser light illuminated on the IRPC, which is essential for the accurate calculation of PCE and short circuit current (*J*_sc_). To accurately determine the effective area of laser beam incident on the cell, we used a laser beam with a spot size that was smaller than the size of the cell. A collimating lens was employed to adjust the spot size of the laser to an area of 0.0176 cm^2^, as determined by a CCD camera. The cell size of the fabricated IRPCs was 0.0414 cm^2^.

The SEM image of the cross-section of the cell was shown in Fig. 2c, which indicated the smoothness of each functional layer. The *J*–*V* curve (under 1 sun illumination) and EQE spectrum of the reference devices are illustrated in Fig. S3. By improving ITO transmittance and utilizing the optical resonance effect, we were able to create an infrared photovoltaic cell with a high PCE of 10.29% under 1550 nm illumination (17.3 mW/cm^2^) and high EQE of 51.04% at 1550 nm as shown in Fig. 2d and e. Remarkably, the champion device achieved a record high PCE of 7.11% under 1 sun illumination (Fig. 2f), and performance parameters of the counterparts reported in recent years was given in Table S1. Compared with the work by the Sargent group in 2018 [23], the *J*_sc_ of our device was 50% higher; this can be attributed to the higher ITO transmittance and optical resonance effect. This high *J*_sc_ is comparable with that achieved in the work by Wang et al. in 2022 [24], but our device exhibited a higher fill factor (FF) and open circuit voltage (*V*_oc_), resulting in higher PCE. Moreover, better passivation of the QD surface and more matching ETL are expected to further improve *V*_oc_ and FF.

**Fig. 3:**
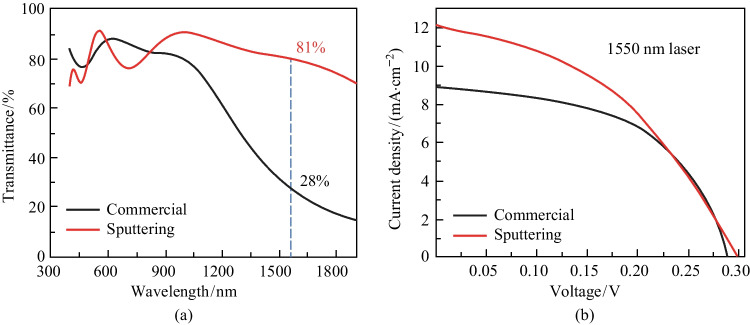
Device optimization through ITO transmittance.** a** Transmittance spectra of commercial ITO and sputtered ITO. **b** *J*–*V* curves of the devices based on commercial and sputtered ITO under 1550 nm illumination (17.3 mW/cm^2^)

Commercial ITO is suitable for full solar spectrum cells with high transmittance in the visible region while exhibiting low transmittance in the infrared region—specifically, lower than 30% at 1550 nm (Fig. 3a) [34–36]. The high concentration of free carriers in commercial ITO results in significant absorption of infrared photons and low infrared transmittance [37]. The infrared transmittance of ITO can be improved by reducing the carrier concentration. We, therefore, improved the ITO transmittance in the infrared region by magnetron sputtering ITO on the glass substrates. By tuning the amount of input oxygen during the sputtering process, the carrier concentration in the sputtered ITO was optimized to balance between transmittance and conductivity. Finally, ITO with transmittance around 81% at 1550 nm (Fig. 3a) was achieved while sacrificing the conductivity to some extent. Figure 3b shows the *J*–*V* curves of commercial ITO based and sputtered ITO-based devices under 1550 nm illumination (17.3 mW/cm^2^). The *J*_sc_ is significantly improved from 8.90 mA/cm^2^ of the commercial-ITO-based devices to 12.14 mA/cm^2^ of the sputtered-ITO-based ones (Table 1). Though sputtered-ITO-based devices exhibited a lower FF value due to lower conductivity, the device PCE increased from 7.89% to 8.77%, with a net increase of 0.88%. These results indicate that the transmittance of ITO is a more crucial factor than its conductivity for the efficiency of IRPCs when operating under 1550 nm laser illumination.

**Table 1:** Performance parameters of CM/SP ITO devices under 1550 nm laser illumination

Device	*J*_sc_/(mA·cm^−2^)	*V*_oc_/V	FF/%	PCE/%
Commercial	8.90	0.287	53.4	7.89
Sputtering	12.14	0.297	42.1	8.77

**Fig. 4:**
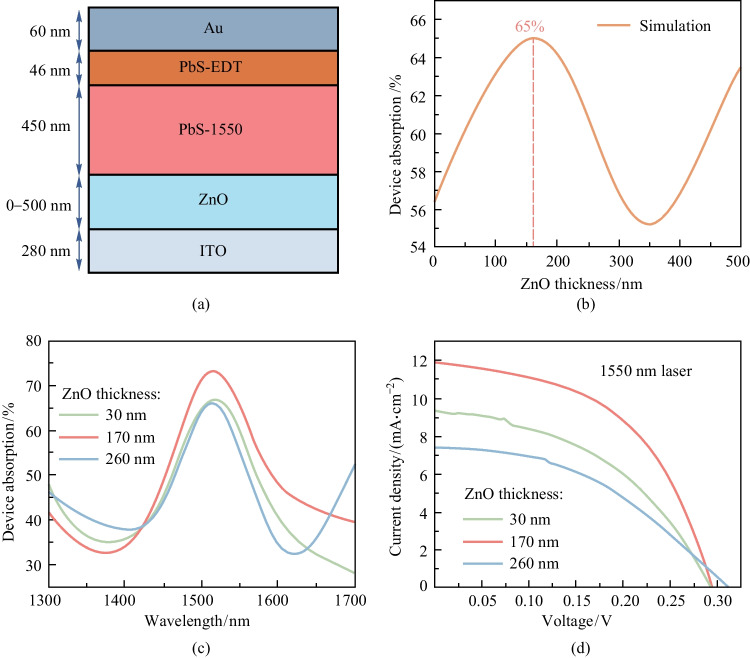
Device optimization through the optical resonance effect. **a** Illustration of the device cross-section with different ZnO thicknesses in the range of 0–500 nm. **b** Calculated absorption at 1550 nm as a function of ZnO layer thickness. **c** Absorption spectra of devices with various thicknesses of the ZnO layer. **d** *J*–*V* curves of devices with various thicknesses of ZnO layer under 1550 nm laser illumination (17.3 mW/cm^2^)

We further enhanced absorption of the IRPC device by utilizing the optical resonance effect for the WOPT systems operating under 1550 nm illumination. The absorption can be increased when the thickness of each layer is well-matched [16, 38]. We used the finite element method (FEM) to calculate the absorption characteristics of the device for different thicknesses of the ZnO layer. The simulation model is illustrated in Fig. 4a, which has 6 layers: Glass (1.1 mm), ITO (280 nm), ZnO (0–500 nm), PbS (450 nm), EDT-PbS (46 nm), and Au (60 nm). Figure 4b shows the calculated absorption of the device as a function of ZnO thickness under 1550 nm illumination. Within the ZnO layer thickness range of 0–500 nm, the device shows a calculated peak absorption of 65% when the ZnO thickness is around 170 nm.

To further verify the simulation results, the ZnO layers with various thicknesses were prepared on ITO substrates by controlling the sputtering time. When the thickness of the ZnO layer ranged from 30 to 260 nm, good agreement between experimental results and calculations was observed. Figure 4c shows that the device with a ZnO layer thickness of 170 nm exhibited more significant absorption enhancement at an incident wavelength of 1550 nm, compared with devices with ZnO layer thickness of 30 and 260 nm. These results indicated that the optical resonance effect was enhanced in devices with a suitable ZnO layer thickness. As expected, the absorption enhancement enabled the increase of current density (Fig. 4d), and devices with a ZnO layer thickness of 170 nm exhibited higher FF (Table 2). We attribute this to the enhanced carrier extraction compared with that for the devices with a 30 nm ZnO layer; as well as better carrier transport in the ETL compared with that for the devices with a 260 nm ZnO layer. The relationship between ZnO depletion region width (*W*_ZnO_) and QD depletion region width (*W*_QD_) can be illustrated as [16]

$${N}_{\mathrm{ZnO}}\times {W}_{\mathrm{ZnO}}={N}_{\mathrm{QD}}\times {W}_{\mathrm{QD}},$$

where *N*_ZnO_ is ZnO dopant concentration and *N*_QD_ is QD dopant concentration. With the increase of ZnO thickness, the QD depletion region is widened and the carrier extraction ability is improved. However, too thick a ZnO layer will make it difficult for extracted carriers to cross the ETL. Due to the significantly improved *J*_sc_ and FF, the PCE of the optimized device was 10.29%, which was 3.24% and 4.63% higher than that of the devices with 30 and 260 nm ZnO layer, respectively. Thus, devices with a ZnO layer thickness of 170 nm demonstrated superior infrared light conversion capability due to the optical resonance effect.

**Table 2:** Performance parameters of devices with various thicknesses of ZnO layer under 1550 nm laser illumination

ZnO devise with various thicknesses/nm	*J*_sc_/(mA·cm^−2^)	*V*_oc_/V	FF/%	PCE/%
30	9.35	0.295	44.2	7.05
170	11.91	0.293	51.0	10.29
260	7.43	0.312	42.3	5.66

**Fig. 5:**
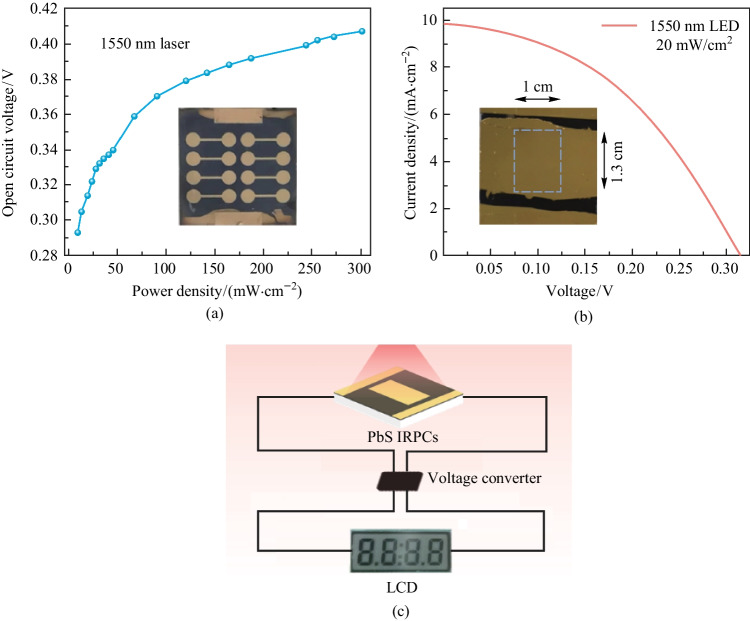
Application in liquid crystal display (LCD) power supply. **a** Open circuit voltage of the IRPC as a function of laser illumination power intensity (10–300 mW/cm^2^). **b** *J*–*V* curve of a device with large active area (1.3 cm^2^) under 1550 nm LED illumination (20 mW/cm^2^). **c** Schematic of powering LCD by infrared PbS CQD PV cell under 1550 nm illumination

We further investigated the performances of the optimized devices under various power intensities of the incident laser light. With the light power intensity changing from 10 to 300 mW/cm^2^ (Fig. S6), we observed that the *J*_sc_ and maximum power output increased along with increasing laser intensity; the rate of increase became lower at high light intensities. Figure 5a shows the variation of open circuit voltage with different incident light power intensities. With laser power intensity varying from 10 to 300 mW/cm^2^, the *V*_oc_ ranges from 0.293 to 0.407 V, which is not enough to power general applications. For instance, operating a liquid crystal display (LCD) through an external power source requires an input voltage of above 3.3 V and an average input power of tens of microwatts. To achieve such voltage, a voltage converter was employed to gather energy from low voltage sources (0.25–0.5 V) and convert it to usable output voltages (3–4 V). After that, we fabricated PbS CQD PV cells with a large active area of 1.3 cm^2^ and the device showed a *V*_oc_ of 0.31 V and output power of 1.7 mW under 1550 nm band light-emitting diode (LED) illumination (20 mW/cm^2^). As shown in Fig. 5c and Fig. S7, the large area PbS CQDs IRPCs were able to power an LCD.

## Conclusions

We developed infrared PbS CQD photovoltaic cells for wireless optical power transfer systems operating at a wavelength of 1550 nm. The PV cells were designed from three photonic aspects: utilizing PbS CQDs with a peak wavelength of 1550 nm, improving ITO transmittance, and enhancing light absorption at 1550 nm by tuning the thickness of the ZnO layer. The devices so produced show enhanced light absorption at 1550 nm when the thickness of the ZnO layer is matched to the resonance condition. The champion device exhibits a high PCE of 10.29% under 1550 nm illumination (17.4 mW/cm^2^), a high EQE of 51% at 1550 nm, and a record high PCE of 7.17% under 1 sun illumination. In addition, we further fabricated large-area PbS cells (1.3 cm^2^) and powered an LCD with the help of a designed voltage converter. Our research provides a new horizon for PbS QD photovoltaic cells working in WOPT systems.

## Experimental section

### Chemicals

Oleylamine (OLA, Aladdin, 90%), oleic acid (OA, Alfa Aesar, 90%), octadecene (ODE, Aladdin, ≥ 90%), 1,2-ethanedithiol (EDT, Aladdin, 97%), acetone (Sinopharm, ≥ 99.5%), ethanol (Sinopharm, ≥ 99.7%), acetonitrile (Sinopharm, ≥ 99.8%), octane (Sinopharm, ≥ 95%), dimethylformamide (DMF, Aladdin, 99.9%), dimethyl sulfoxide (DMSO, Innochem, 99.8%), butylamine (BTA, Aladdin, 98%), 4-(aminomethyl)pyridine (4-AMPY, Aladdin, 99.9%), lead oxide (PbO, Aladdin, 99.999%), hexamethyldisilathiane (TMS, Macklin, 95%), lead chloride (PbCl_2_, Aladdin, 99.99%), lead bromide (PbBr_2_, Advanced Election Technology, ≥ 99.99%) and lead iodide (PbI_2_, Advanced Election Technology, 99.999%) were used without further purification.

### Deposition of ITO and ZnO films

The magnetron sputtering instrument was JCP500 with a high vacuum multi-target system. The ITO and ZnO (99.9% purity) sputtering targets were purchased from Zhongnuo New Material Company. To deposit the ITO layer on the SiO_2_ substrate, the sputtering power was adjusted to 100 W and lasted for 20 min at room temperature. Mixed oxygen and argon, O_2_:Ar = 0.3:33, were used as the sputtering gas. The chamber pressure was around 0.1 Pa during the sputtering process and the sputtering rate was about 2 Å/s. The deposition of the ZnO layer on ITO followed a similar process, but with the gas ratio of O_2_:Ar = 1:99, and the chamber pressure was around 4 Pa. The sputtering power was adjusted to 180 W and lasted for 30 min at room temperature.

### Synthesis of PbS QDs

PbS QDs with excitonic peaks at around 900 nm (small size) and 1550 nm (large size) were synthesized by the hot injection and cation exchange methods, respectively. For the small size PbS QDs, a mixture of lead oxide (2.25 g), oleic acid (8 mL), and octadecene (20 mL) was loaded in a 150 mL flask under an N_2_ atmosphere, then degassed and gradually heated and then maintained at 100 °C for 1.5 h. After that, 700 μL TMS in 5 mL octadecene was injected into the flask and the mixture was vigorously stirred for 55 s. After washing, the separated QDs were re-dispersed in octane with a concentration of 40 mg/mL for device fabrication. For large-size QDs, ZnS QDs were used as a sulfur source; 1.946 g (0.012 mol) of PbCl_2_ and 40 mL OLA were loaded in a 250 mL three-neck flask, and the mixture was degassed for 15 min under room temperature. The mixture's temperature was then heated to 140 °C under N_2_ atmosphere and maintained for 30 min to form the lead precursor (Pb-OLA). Subsequently, the lead precursor was set to 90 °C for the injection of the high-concentration ZnS QDs (diluted in 5 mL ODE). Then the temperature was maintained for 30 s for rapid nucleation. The next step was injecting ZnS QDs into the PbCl_2_ precursor solution to obtain PbS QDs by the combination of rapid injection (nucleation) and droplet injection (growth). The whole growth process lasted for ~ 55 min and the temperature of the reaction system was raised from 90 to 120 °C gradually during the ZnS QD dropping process. Then the flask. was quenched using a water bath and *n*-hexane (40 mL) was injected into the reaction flask at 70 °C; 40 mL of OA was injected once the temperature fell below 40 °C. The reaction mixture was stirred for 10 min. The raw solution was then centrifuged to remove the unreacted PbCl_2_ and the supernatant was subjected to purification using acetone as the antisolvent.

### Device fabrication

The following process was conducted in a nitrogen atmosphere glove box. First, the control ligand was dissolved in 10 mL DMF solvent at the molar ratio of PbI_2_ (1.229 g):PbBr_2_ (0.428 g) (2.3:1). Then 10 mL PbS QD solution with a concentration of 10 mg/mL and *n*-octane as solvent was added to the centrifuge tube containing the above control ligand for ligand exchange. After three repetitions of solution-phase ligand exchange in the DMF and octane solvent system, the 380 mg/mL PbS-IBr (PbI_2_ and PbBr_2_ capped PbS QDs) in DMF:DMSO:BTA:4-AMPY (50:30:17:3) solvent was spin-coated onto ZnO film at 2500 r/min for 45 s and then annealed at 90 °C for 10 min. The absorber film cooled down naturally to below 40 °C. Then, two layers of EDT-treated PbS QDs (excitonic peak at 890 nm) were grown to act as the whole transport layer. Finally, the Au layer with a thickness of 60 nm was deposited by thermal evaporation, to act as the upper electrode. The effective area of the prepared QD solar cell was 0.0706 cm^2^.

### Characterization of materials and devices

The optical absorption spectra of QDs were measured by a Shimadzu UV-3600 Plus spectrophotometer. The absorption spectra of PbS QDs film and the devices were collected using a spectrophotometer (PerkinElmer instrument, Lambda 950). The scanning electron microscopy (SEM) images were obtained using FEI Nova Nano SEM 450. The ZnO film crystallization was tested by X-ray diffractometer (XRD) with Cu Kα radiation (Philips, X pert pro-MRD, Netherlands). EQE of PbS QD PV cells was measured using a Quantum Efficiency Measurement Instrument QE-R (Enlitech Co., Ltd). The current density–voltage characteristics were recorded with a Keithley 2400 digital source meter under simulated solar light illumination (AM 1.5, 100 mW/cm^2^) or under 1550 nm laser illumination in the air at room temperature.

### FEM simulation

Calculated absorption of the devices as a function of ZnO thickness at 1550 nm was simulated with commercial FEM software (COMSOL). The thickness of each layer in the simulation model was: Glass (1.1 mm), ITO (280 nm), ZnO (30–260 nm), EDT-PbS (46 nm), Au (60 nm), and PbS (450 nm). The incident light was a planar wave. The periodical boundary condition and perfectly matched layer were applied for the simulation of multi-layer films.

## Supplementary Information

Below is the link to the electronic supplementary material.

Supplementary file1 (PDF 342 KB)

## Data Availability

The data that support the findings of this study are available from the corresponding author, upon reasonable request.
