# Supplementary material for: Optical engineering of infrared PbS CQD photovoltaic cells for wireless optical power transfer systems
Source: Front Optoelectron. 2023 Jun 15;16(1):15. doi: 10.1007/s12200-023-00069-0 (PMC10271996; doi:10.1007/s12200-023-00069-0)
Supplement: Supplementary file 1 — Supplementary file1 (PDF 342 KB) [file 12200_2023_69_MOESM1_ESM.pdf]

Supporting Information

Optical engineering of infrared PbS CQD photovoltaic cells for wireless optical power transfer system

Mengqiong Zhu[1#], Yuanbo Zhang[2#], Shuaicheng Lu[2], Zijun Wang[2], Junbing Zhou[1], Wenkai Ma[2], Ruinan Zhu[1], Guanyuan Chen[1], Jianbing Zhang[2], Liang Gao[2], Jiancan Yu [1*], Pingqi Gao[1], Jiang Tang[2*]

[1]School of Materials, Shenzhen Campus of Sun Yat-sen University, Shenzhen 518107, China

[2]Wuhan National Laboratory for Optoelectronics (WNLO) and School of Optical and Electronic Information, Huazhong University of Science and Technology (HUST), Wuhan 430074, China

# These authors contributed equally to this work

J. Yu: yujc3@mail.sysu.edu.cn; J. Tang: jtang@mail.hust.edu.cn

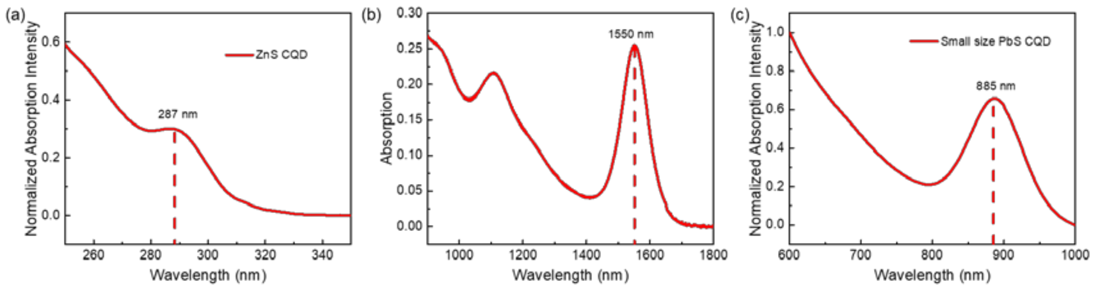

Figure S1 (a) The absorption spectrum of ZnS QD, (b) 1550 nm PbS QD (synthesized by cation exchange method), (c) and 885 nm PbS QDs (synthesized by hot injection method).

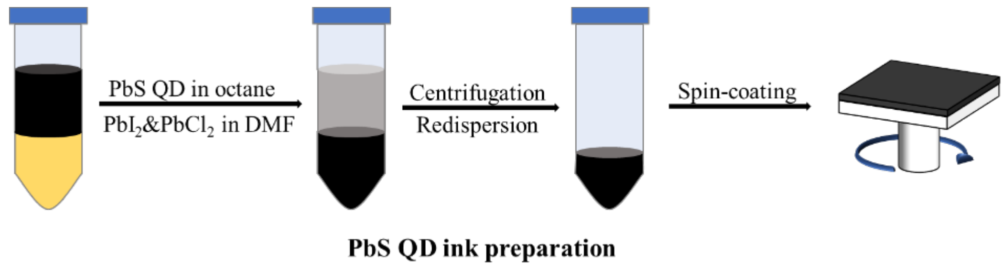

Figure S2 PbS QD ink preparation

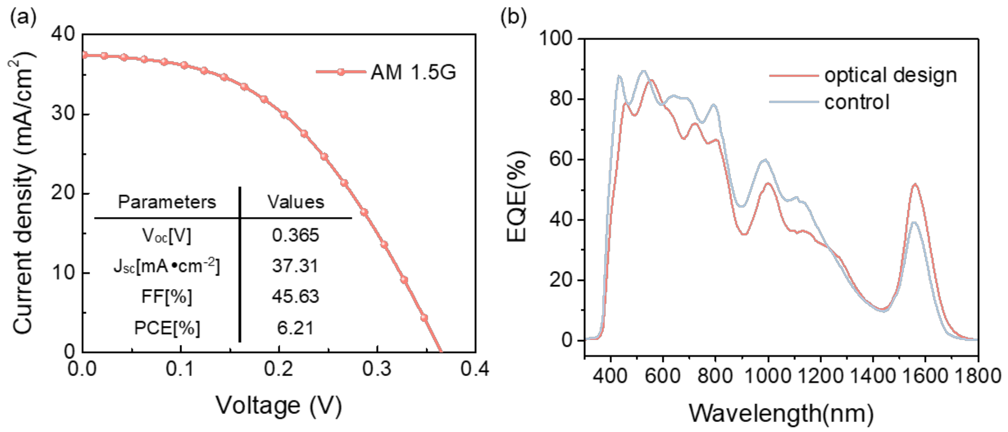

Figure S3. (a) *J-V* curves and performance parameters of control device under AM 1.5G solar illumination. (b) EQE spectrum of champion device with optical design and control device.

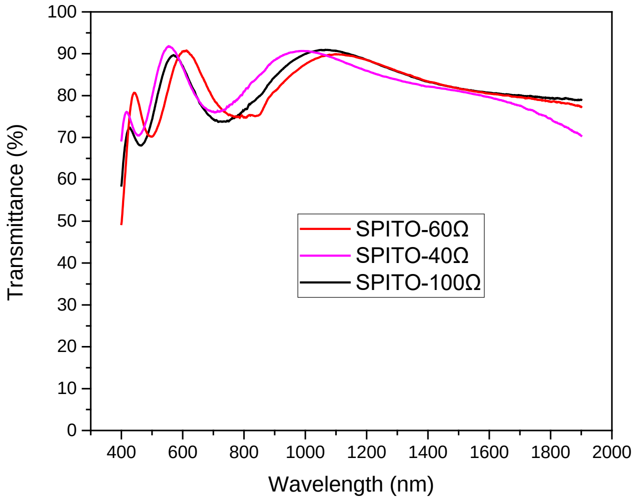

Figure S4 Sputtering ITO transmission.

Table S1. Performance of infrared solar cells based on excitonic 1550 nm PbS CQDs.

Year	1st exciton peak (nm)	Illumination	J_{sc}(mA/cm^2)	V_{oc}(V)	FF(%)	PCE(%)
2018	1550	AM 1.5G	27.8	0.41	50	5.8
2022	1560	AM 1.5G	39.8	0.341	41.1	5.56
This work	1550	AM 1.5G	37.65	0.395	48.17	7.17

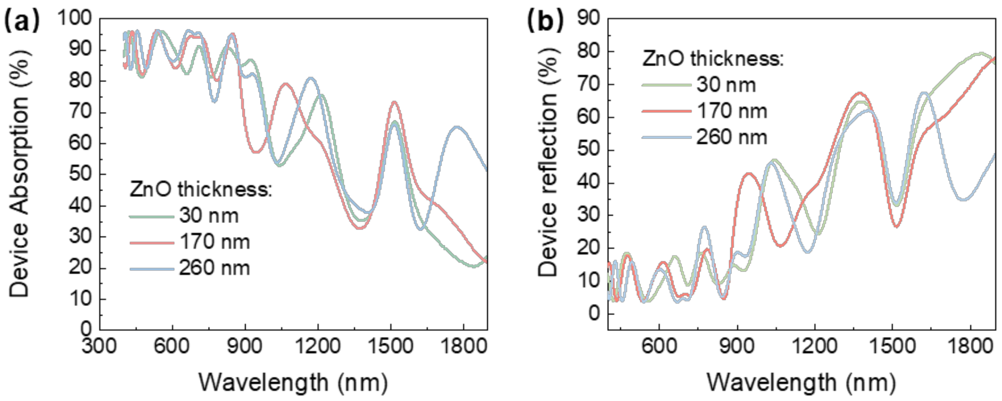

Figure S5. The absorption (a) and reflection (b) spectrum of devices with various thicknesses of ZnO layer.

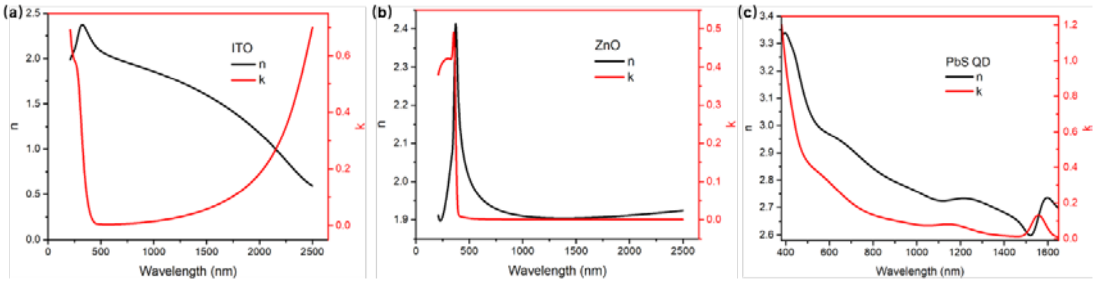

Figure S6. Wavelength dependence of the refractive index (n) and extinction coefficient (k) of ITO (a), ZnO (b), and PbS QD (c).

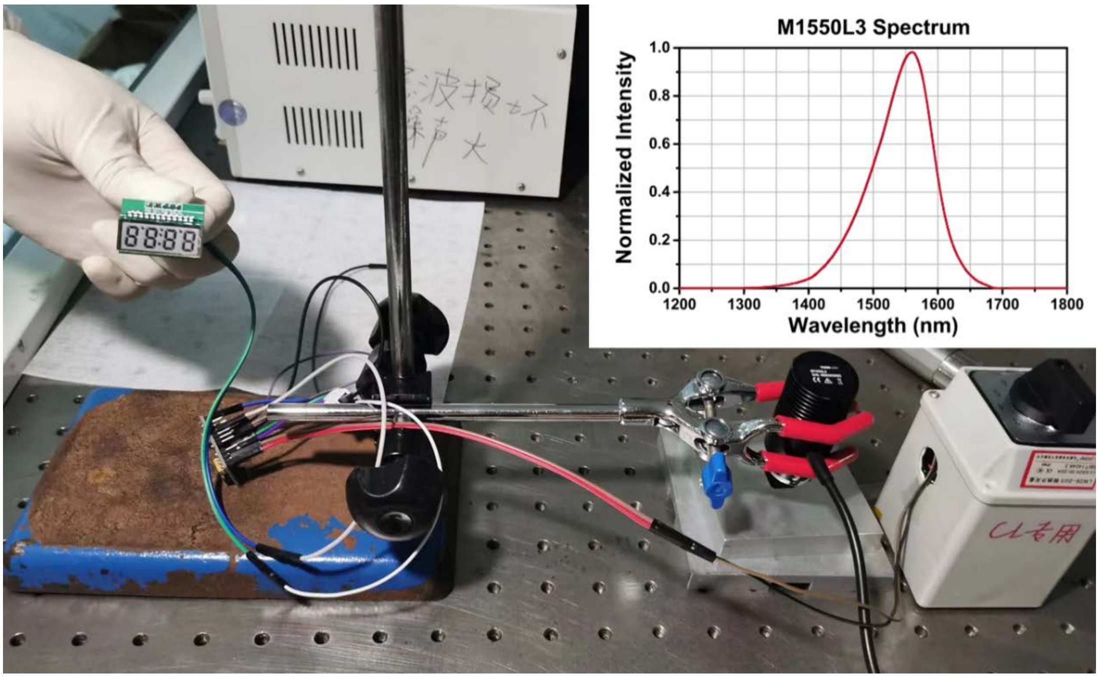

Figure S7. Demonstration of lighting an LCD through the PbS CQD IRPCs under 1550 nm illumination. Inset, the spectrum of 1550 nm LED.
